# Supplementary material for: Content-rich biological network constructed by mining PubMed abstracts
Source: BMC Bioinformatics. 2004 Oct 8;5:147. doi: 10.1186/1471-2105-5-147 (PMC528731; doi:10.1186/1471-2105-5-147)
Supplement: Additional File 5 — The original Chilibot query results of the term "long-term potentiation (LTP)" and 22 other terms, limiting the latest references analyzed to the years 1990, 1995, 2000, and 2004. [file 1471-2105-5-147-S5.bz2 › chilibotAdditionalFile5/ltp2004/html/TAU_SYNAPTOPHYSIN.html]

 


 **TAU** and **SYNAPTOPHYSIN** 
  
Found 69 abstracts in PubMed,  **30 abstracts were retrieved and analyzed**.  


---

 Search Google  |
 PDF files only 
|  EDU domain only 

---

**Interactive relationship** (e.g. stimulation, inhibition, etc)

- We found that there is a graded, progressive, decrease of  **synaptophysin**  message expressed by single neurons related to immunohistochemical markers of  **tau**  status,.  Ref: 12025941 J Neuropathol Exp Neurol, 2002
- In the hippocampal slice model,  **tau**  deposits and amyloidogenic fragments induced by the lysosomal inhibitor chloroquine were accompanied by disrupted microtubule integrity and by corresponding declines in postsynaptic glutamate receptors and the presynaptic marker  **synaptophysin** .  Ref: 12769185 J Neuropathol Exp Neurol, 2003
- When such activated microglia were placed in coculture with primary neocortical neurons, a significant increase in the phosphorylation of neuronal  **tau**  was accompanied by a decline in  **synaptophysin**  levels.  Ref: 12629164 J Neurosci, 2003
- Here we report that nanomolar thrombin induced rapid  **tau**  hyperphosphorylation and aggregation in murine hippocampal neurons via protease activated receptors, which was followed by delayed  **synaptophysin**  reduction and apoptotic neuronal death.  Ref: 12821672 J Biol Chem, 2003

- :-)

  **Parallel relationship** (e.g. studied together, co-existance, homology, etc.)

  - In this study we analyse the hippocampal expression patterns of hyperphosphorylated  **tau** , paired helical filament PHF related protein, beta amyloid and  **synaptophysin**  in a group of Alzheimer s disease AD sufferers with and without additional pathology.  Ref: 10912925 Acta Neuropathol (Berl), 2000
  - As a direct test of the requirement for IL 1 in  **tau**  phosphorylation and  **synaptophysin**  expression, IL 1 actions in neuron microglia cocultures were manipulated.  Ref: 12629164 J Neurosci, 2003
  - Ganglioneuromatosis was characterized by diffuse Schwann cell hyperplasia expressing S100 protein in close contact to nerve fibers expressing neurofilament,  **tau**  protein,  **synaptophysin** , PGP9.5 and to ganglion cells expressing c Ret.  Ref: 12658132 Gastroenterol Clin Biol, 2003
  - These neurons displayed extensive processes, exhibited immunoreactivity for mature neuron specific markers such as  **tau**  and  **synaptophysin** , and fired action potentials upon current injection.  Ref: 10679769 J Neurosci Res, 2000
  - In addition to the routine examination including methenamine Bodian staining, an immunohistochemical study was performed, using antibodies to amyloid beta protein,  **tau** , ubiquitin, glial fibrillary acidic protein GFAP,  **synaptophysin** , and Ki MIP a marker of the microglial and perivascular cells.  Ref: 9600680 J Neurol Sci, 1998
  - METHODS We have studied, by immunohistochemistry and immunoblotting, the presence of neuronal cytoskeletal antigens class III beta tubulin isotype beta III, microtubule associated proteins MAP2, MAP1B and  **tau** , and different neurofilament NF protein components, and  **synaptophysin**  in comparison with the glial fibrillary acidic protein GFAP and S 100 protein in the PC12 cell line.  Ref: 9517852 Anat Rec, 1998
  - Stress responsive MAP kinase pathways were activated in the brain of the Tg2576 PS1 P264L AD model, and this activation was coincident with the age dependent increase in amyloid deposition,  **tau**  phosphorylation, and loss of  **synaptophysin** .  Ref: 11978814 J Neurosci, 2002
  - Some effects of microglial activation on neurons appear to be mediated by activation of p38 mitogen activated protein kinase p38 MAPK, because activated microglia stimulated p38 MAPK phosphorylation in neurons, and an inhibitor of p38 MAPK reversed the influence of IL 1beta on  **tau**  phosphorylation and  **synaptophysin**  levels.  Ref: 12629164 J Neurosci, 2003
  - Interleukin 1 mediates pathological effects of microglia on  **tau**  phosphorylation and on  **synaptophysin**  synthesis in cortical neurons through a p38 MAPK pathway.  Ref: 12629164 J Neurosci, 2003
  - Conversely, the differentiating neurons derived from populations and clonal lines of MSCs expressed the specific markers beta III tubulin,  **tau** , neurofilament M, TOAD 64, and  **synaptophysin**  de novo.  Ref: 12205683 J Neurosci Res, 2002
  - The developmentally controlled expression of skeletal muscle specific genes coding for myf5, myogenin, myoD and myf6, alpha 1 subunit of the L type calcium channel, cell adhesion molecule M cadherin, and neuron specific genes encoding the 6 60, and 200 kDa neurofilament proteins, synaptic vesicle protein  **synaptophysin** , brain specific proteoglycan neurocan, and microtubule associated protein  **tau**  was demonstrated by RT PCR analysis.  Ref: 9521839 Exp Cell Res, 1998
  - An immunohistochemical study was performed with antibodies directed against S100 protein,  **synaptophysin** , PGP9.5, neurofilament,  **tau**  protein, c Kit and c Ret.  Ref: 12658132 Gastroenterol Clin Biol, 2003
  - IL 1 receptor antagonist IL 1ra as well as anti IL 1beta antibody attenuated the influence of activated microglia on neuronal  **tau**  and  **synaptophysin** , but anti TNFalpha antibody was ineffective.  Ref: 12629164 J Neurosci, 2003
  - There were several subsets of neurites that were argyrophilic or immunoreactive against antibodies to either phosphorylated  **tau**  protein, phosphorylated neurofilaments, ubiquitin, or  **synaptophysin** .  Ref: 10378375 Acta Neuropathol (Berl), 1999
  - **Synaptophysin** , neurofilaments and  **tau**  labeling was found in cells forming the pineocytomatous rosettes.  Ref: 9600600 Acta Neuropathol (Berl), 1998
  - Double labeling studies showed that all three of these neuropathological structures also contained ubiquitin,  **synaptophysin** , and neurofilament but not  **tau**  immunoreactivity.  Ref: 9466562 Am J Pathol, 1998
  - Tissue sections were immunostained with vimentin, CD34, desmin, specific smooth muscle actin, S100 protein, neuron specific enolase, PGP9.5, neurofilament, bcl 2 oncoprotein,  **synaptophysin** , chromogranin A, c kit, and  **tau** .  Ref: 11727254 Hum Pathol, 2001
  - However, at 39 degrees C, the cells produced dendritic, neuronal like processes and elevated levels of NF68 and MAP2, as well as the neuronal markers  **synaptophysin** , neurone specific enolase, and low levels of  **tau** , all determined by western blotting and immunofluorescent staining.  Ref: 10537044 J Neurochem, 1999
  - Cultures treated for up to 5 weeks with epidermal growth factor, fibroblast growth factor 2, retinoic acid, and nerve growth factor displayed neuron like cellular processes and expressed neuronal markers, including the neuronal nuclear antigen NeuN, microtubule associated protein 2,  **tau** ,  **synaptophysin** , alpha 1A and alpha 1B calcium channel subunits, NR2A glutamate receptor subunits, and gamma aminobutyric acid.  Ref: 14637082 Exp Neurol, 2003
  - Compared with other neuronal markers, the positive ratio of alpha synuclein was not as high as  **synaptophysin** , microtubule associated protein 2, neuron specific enolase and  **tau** , but it was higher than neurofilament and chromogranin A.  Ref: 10672322 Acta Neuropathol (Berl), 2000
  - On immunstaining some of the OLCs were positive for  **synaptophysin**  and negative for glial fibrillary acidic protein GFAP, glucocerebroside,  **tau**  and MAP 2.  Ref: 9832257 Clin NeuropatholClin Neuropathol, 2000
  - The neuronal phenotype of PC12 cells was confirmed by staining for MAP2,  **tau** , and NF proteins, as well as for  **synaptophysin** .  Ref: 9517852 Anat Rec, 1998
  - Unlike the epithelial parental Panc 1 cells, these cells exhibited the appearance of a neuroendocrine like phenotype with extensive branch like processes, and marked cytoplasmic and cytoskeletal immunostaining for  **tau**  2,  **synaptophysin** , and chromogranin A.  Ref: 9569027 Oncogene, 1998
  - Ultrastructural and double immunolabeling analysis confirmed that most plaques were mature and contained dystrophic neurites immunoreactive with antibodies against APP,  **synaptophysin** , neurofilament and  **tau** .  Ref: 11746377 J Neurosci Res, 2001
